# Molecular profiling of circulating tumor cells links plasticity to the metastatic process in endometrial cancer

**DOI:** 10.1186/1476-4598-13-223

**Published:** 2014-09-27

**Authors:** Lorena Alonso-Alconada, Laura Muinelo-Romay, Kadri Madissoo, Antonio Diaz-Lopez, Camilla Krakstad, Jone Trovik, Elisabeth Wik, Dharani Hapangama, Lieve Coenegrachts, Amparo Cano, Antonio Gil-Moreno, Luis Chiva, Juan Cueva, Maria Vieito, Eugenia Ortega, Javier Mariscal, Eva Colas, Josep Castellvi, Maite Cusido, Xavier Dolcet, Hans W Nijman, Tjalling Bosse, John A Green, Andrea Romano, Jaume Reventos, Rafael Lopez-Lopez, Helga B Salvesen, Frederic Amant, Xavier Matias-Guiu, Gema Moreno-Bueno, Miguel Abal, on behalf of ENITEC Consortium

**Affiliations:** Translational Medical Oncology; Health Research Institute of Santiago (IDIS), SERGAS, Trav. Choupana s/n 15706, Santiago de Compostela, Spain; Department of Obstetrics and Gynecology, Haukeland University Hospital, Bergen, Norway; Department of Clinical Science, Centre for Cancer Biomarkers, University of Bergen, Bergen, Norway; Departament of Biochemistry, Universidad Autónoma de Madrid (UAM), Instituto de Investigaciones Biomédicas “Alberto Sols” (CSIC-UAM), IdiPAZ, Madrid, Spain; Department of Pathology, Haukeland University Hospital, Bergen, Norway; Department of Women’s and Children’s Health, Institute of Translational Medicine, University of Liverpool, Liverpool Women’s Hospital, Crown Street, Liverpool, UK; Division of Gynecologic Oncology, Department of Oncology, KU Leuven, B-3000 Leuven, Belgium; Research Unit in Biomedicine and Translational and Pediatric Oncology, Vall d’Hebron Research Institute and Hospital and Universitat Autònoma de Barcelona, Barcelona, Spain; Hospital MD Anderson Cancer Centre Madrid, Madrid, Spain; Department of Pathology and Molecular Genetics and Research Laboratory, Hospital Universitari Arnau de Vilanova, University of Lleida, IRBLLEIDA, Lleida, Spain; Clinical and Surgical Gynaecology R&D + I, Institut Universitari Dexeus, Barcelona, Spain; University Medical Center Groningen, Department of Gynecologic Oncology, University of Groningen, P.O. Box 30.001, 9700 RB Groningen, The Netherlands; Department of Pathology, Leiden University Medical Center, Leiden, The Netherlands; GROW: School for Oncology and Developmental Biology, Department of Obstetrics and Gynaecology, Maastricht University Medical Centre, Maastricht, The Netherlands; Departament de Ciencies Basiques, Universitat Internacional de Catalunya, Barcelona, Spain

**Keywords:** High-risk endometrial carcinomas, Circulating tumor cells, Epithelial to mesenchymal transition, Stem cell, ETV5

## Abstract

**Background:**

About 20% of patients diagnosed with endometrial cancer (EC) are considered high-risk with unfavorable prognosis. In the framework of the European Network for Individualized Treatment in EC (ENITEC), we investigated the presence and phenotypic features of Circulating Tumor Cells (CTC) in high-risk EC patients.

**Methods:**

CTC isolation was carried out in peripheral blood samples from 34 patients, ranging from Grade 3 Stage IB to Stage IV carcinomas and recurrences, and 27 healthy controls using two methodologies. Samples were subjected to EpCAM-based immunoisolation using the CELLection™ Epithelial Enrich kit (Invitrogen, Dynal) followed by RTqPCR analysis. The phenotypic determinants of endometrial CTC in terms of pathogenesis, hormone receptor pathways, stem cell markers and epithelial to mesenchymal transition (EMT) drivers were asked. Kruskal-Wallis analysis followed by Dunn’s post-test was used for comparisons between groups. Statistical significance was set at p < 0.05.

**Results:**

EpCAM-based immunoisolation positively detected CTC in high-risk endometrial cancer patients. CTC characterization indicated a remarkable plasticity phenotype defined by the expression of the EMT markers *ETV5, NOTCH1, SNAI1, TGFB1, ZEB1* and *ZEB2*. In addition, the expression of *ALDH* and *CD44* pointed to an association with stemness, while the expression of *CTNNB1, STS, GDF15, RELA, RUNX1, BRAF* and *PIK3CA* suggested potential therapeutic targets. We further recapitulated the EMT phenotype found in endometrial CTC through the up-regulation of *ETV5* in an EC cell line, and validated in an animal model of systemic dissemination the propensity of these CTC in the accomplishment of metastasis.

**Conclusions:**

Our results associate the presence of CTC with high-risk EC. Gene-expression profiling characterized a CTC-plasticity phenotype with stemness and EMT features. We finally recapitulated this CTC-phenotype by over-expressing *ETV5* in the EC cell line Hec1A and demonstrated an advantage in the promotion of metastasis in an in vivo mouse model of CTC dissemination and homing.

**Electronic supplementary material:**

The online version of this article (doi:10.1186/1476-4598-13-223) contains supplementary material, which is available to authorized users.

## Introduction

Endometrial carcinomas, the most common tumors of the female genital tract, are usually diagnosed at an early stage with uterine-confined disease and an overall favorable prognosis. However, up to 20% of endometrial carcinomas present as aggressive neoplasms such as high-grade or deeply invasive lesions, at substantial risk of recurrence and death [1]. Propagation of aggressive tumor cells from the primary lesion is a key event in the process of metastasis and a challenge in oncology. In endometrial cancer, myometrial infiltration, lymph node involvement, and lymphovascular space invasion are current clinical parameters defining the probability of recurrent disease. Nevertheless, early dissemination of tumor cells is usually undetectable in patients by conventional histopathological examination or by standard imaging techniques. Recently, immunocytochemical and molecular assays have been developed for the specific detection of metastatic tumor cells at a cellular level in lymph nodes, peripheral blood or bone marrow, prior to the manifestation of metastasis. Tumor-cell dissemination can proceed at an early stage of tumor development [2], and detecting circulating tumor cells (CTC) has clinical value in the monitoring and the outcome of metastatic disease. CTC analysis represents an attractive candidate for liquid biopsy in cancer [3]. Clinically, the presence of CTC above a threshold may have a significant adverse impact on survival. Likewise, changes on CTC quantification during treatment can reflect prognostic significance, the future challenge being whether treatment decision-making should be impacted by CTC levels [4].

The increasing interest in CTC at the clinical setting is resulting in the development of a number of innovative technologies that include immunoenrichment, microfluidics and filtration devices, combined with semiautomated microscopy or PCR-based detection systems [5]. We have recently demonstrated that the combination of CTC EpCAM-based immunoisolation, followed by accurate extraction and pre-amplification of RNA from very small number of CTC, provided with a highly sensitive approach to profiling the metastatic tumor cell population in a group of colorectal cancer patients [6]. In the present study, we adopted a similar approach in high-risk EC patients. CTC immunoisolation plus profiling of a number of genes related to key events in the process of metastasis in EC provided us with an overview of the biology of endometrial CTC. In addition to analyze in immunoisolated CTC the expression of a number of genes involved in signaling pathways related to EC, hormone pathways, stem cell features and epithelial to mesenchymal transition (EMT) markers, we evaluated the efficiency of CTC quantification and its correlation with clinical parameters.

## Results

### Assessment of CTC in the blood samples from high-risk EC patients

**Figure 1:**
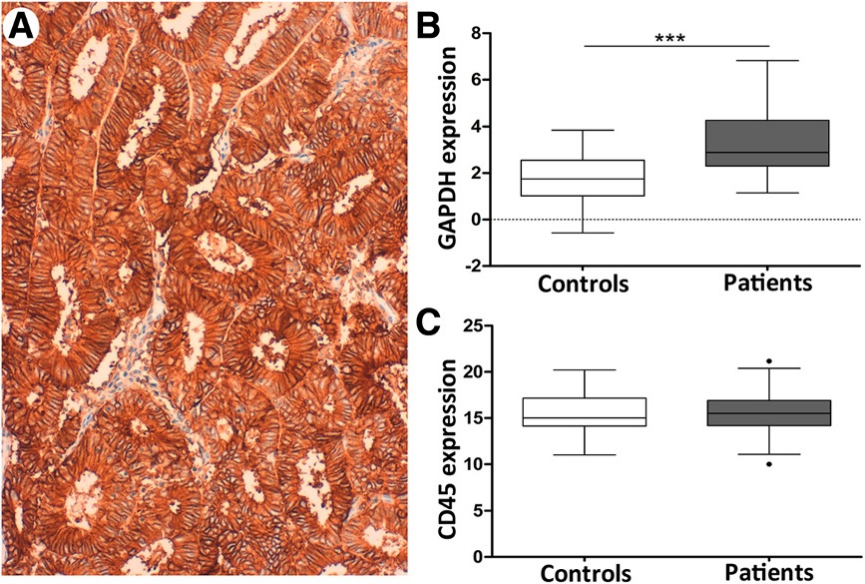
**Immunoisolation of CTC in high-risk EC patients. (A)** Representative primary carcinoma of a patient included in the study, demonstrating positivity for membrane EpCAM staining in epithelial tumor cells. **(B)** *GAPDH* expression levels normalized to *CD45* in CTC isolated from the group of controls (white box, n = 27), as background of unspecific immunoisolation, and from the group of high-risk EC patients (grey box, n = 34) (Mann–Whitney test, ***p < 0.001). **(C)** *CD45* expression levels in CTC isolated from controls and patients; similar expression denoted equivalent degree of unspecific immunoisolation.

Immunoisolation of CTC from peripheral blood samples has been performed with magnetic beads coated with EpCAM antibodies. We thus first confirmed the positivity for EpCAM expression in the corresponding primary carcinomas of a representative sample of patients included in the study (Figure 1A). Secondly, we investigated the presence and quantified the amounts of CTC in a series of 34 EC patients ranging from Grade 3 Stage IB carcinomas to metastatic Stage IV carcinomas and recurrences (see global clinical descriptions in Table 1). CTC were immunoisolated with EpCAM-dynabeads from EDTA-BD Vacutainer 7.5 ml blood collection tube. Upon RNA extraction and pre-amplification, we evaluated the expression levels of *GAPDH* as a marker of cellularity, which includes both CTC and unspecific blood cells, normalized to the background of *CD45* expression as specific marker for cells of hematopoietic origin [7]. As shown, *GAPDH* levels were significantly higher in the group of patients compared to controls (Figure 1B; with 30 high-risk EC patients below control upper threshold and 3 controls above lower patients’ threshold), while *CD45* did not present differences between both groups (Figure 1C), indicating (i) the presence of an extra population of cells isolated from the blood of high-risk EC patients and (ii) the unspecific background resulting from the process of immunoisolation was similar in the group of patients and controls. The presence of CTC in high-risk endometrial cancer patients was further confirmed with the technology that received to date Food and Drug Administration (FDA) clearance for the monitoring of metastatic breast, colorectal, and prostate cancer, the CellSearch System (Janssen Diagnostics, SouthRaritan, NJ, USA), which combines immunoenrichment and immunofluorescence for the detection of CTC [8–10] (Additional file 1). Globally, these results demonstrated in parallel the presence of CTC in high-risk EC patients.

**Table 1:** **Clinical and pathologic characteristics of high-risk EC patients included in the gene-expression profiling of CTC**

Feature	n (%)	Feature	n (%)
Age (years)			
Mean	69		
FIGO Stage		Lymphovascular invasion	
I	15 (44.15)	Positive	6 (17.6)
II	2 (5.8)	Negative	15 (44.1)
III	11 (32.35)	Unknown	13 (38.2)
IV	6 (17.64)	Lymph node metastasis	
Histology		Positive	12 (35.3)
Endometrioid	19 (55.9)	Negative	20 (58.8)
Serous	10 (29.4)	Unknown	2 (5.9)
Clear cell	5 (14.7)	Recurrence	
Grade		No	18 (52.9)
Well differentiated (G1)	4 (11.8)	Yes	15 (44.1)
Moderately differentiated (G2)	6 (17.6)	Unknown	1 (2.9)
Poorly differentiated (G3)	21 (61.8)	First treatment	
Unknown	3 (8.8)	Radiotherapy	4 (11.8)
Myometrial invasion		Chemotherapy	7 (20.6)
<50%	13 (38.2)	None	17 (50)
>50%	18 (52.9)	Unknown	6 (17.6)
Unknown	3 (8.8)		

Unknown values were considered as missing and therefore excluded from the statistical analysis.

### Gene-expression analysis in immunoisolated CTC from EC patients highlights a plasticity phenotype

Once we confirmed their presence, we explored the gene-expression profile of CTC in the samples from high-risk EC patients upon EpCAM-based immunoisolation and RNA extraction and pre-amplification. For this, we analyzed genes of signaling pathways previously reported to be related to EC (*BRAF, CTNNB1, ERBB2, FGFR2, GDF15, IDO, MTOR, P53, PIK3CA, PTEN, PTGS2, RUNX1, RELA, STMN1, TERT, VIL1, ZWINT*), hormone pathways (*CYP19, ESR1, ESR2, GPER, HSD17B1, PGR, STS, TFF1*), stem cell features (*ALDH, CD133, CD44*), and EMT related markers (*ETV5, LOXL2, NOTCH1, SNAI1, TGFB1, ZEB1, ZEB2*) (Additional file 2). Initially, where in a preliminary set of 6 patients and 6 controls no amplified signal could be observed either in patients or in controls probably due to non-detectable levels of expression, genes were excluded from further evaluation. This primary screen resulted in exclusion of *CD133, GPER, HSD17B1, PGR*, and *TERT* (data not shown).

**Figure 2:**
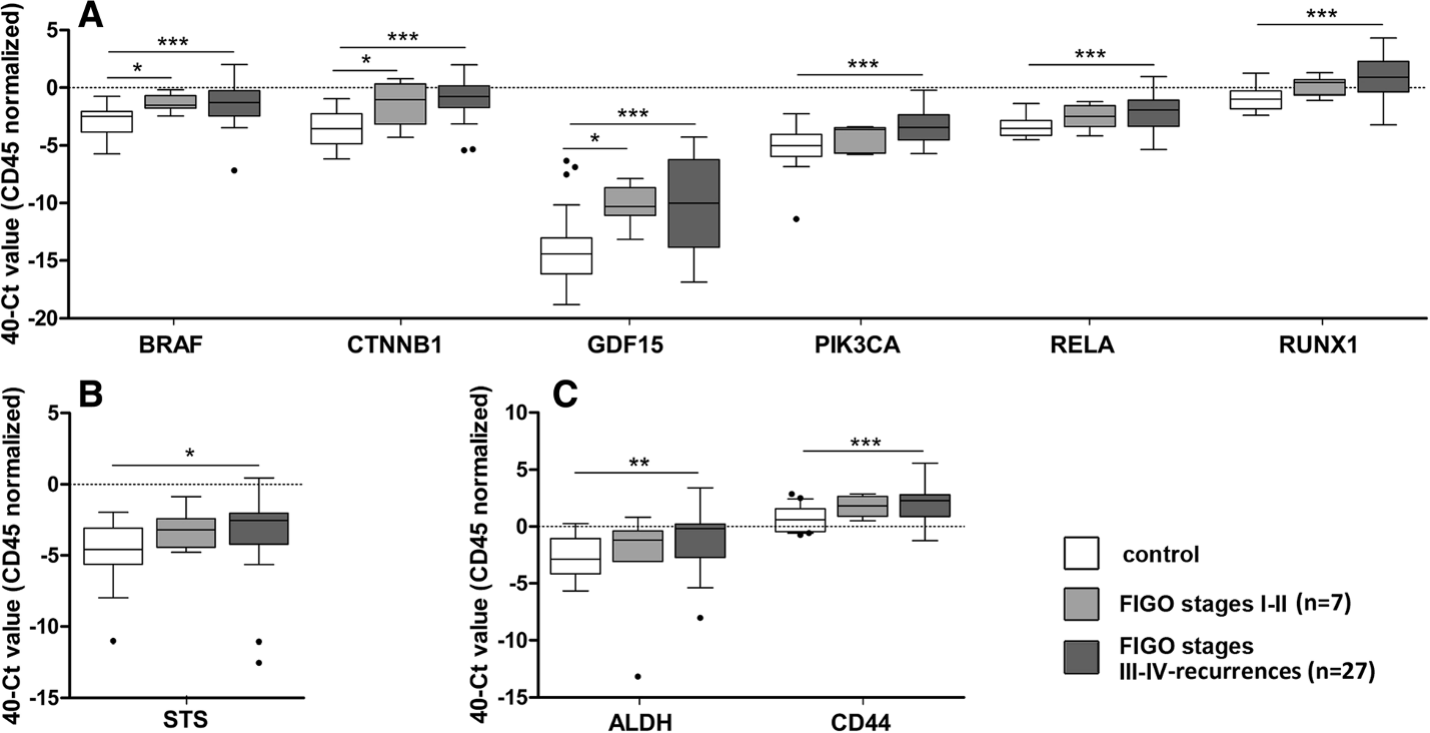
**Gene expression profiling in endometrial CTC. (A)** Significant expression levels of genes involved in signaling pathways reported altered in EC, **(B)** hormone pathways and **(C)** stem cell features, in CTC from high-risk EC patients compared to the background of unspecific immunoisolation. White boxes represent the gene expression levels in the group of healthy controls, light grey boxes those corresponding to FIGO Stages I and II EC patients while dark grey boxes those corresponding to FIGO Stages III-IV EC patients and recurrences. (Kruskal-Wallis test, *p < 0.05; **p < 0.01; ***p < 0.001).

We next analyzed the expression levels of the remaining genes in the whole set of patients and controls, and identified those genes with a significant expression in CTC from the group of patients compared to the background of unspecific isolation from the controls. These genes are considered to characterize the population of CTC in EC. We further grouped those high-risk patients presenting FIGO Stages I-II and those with FIGO Stages III-IV and recurrences, as stages presenting no or local cell tumor spread and those presenting systemic dissemination, respectively. Overall, major significances were found between the group of healthy controls and FIGO Stages III-IV and recurrences, consistent with a gradual increased presence of CTC as the disease disseminates; modest increases in a reduced number of genes in FIGO Stages I-II that might suggest the presence of CTC in early stages has to be interpreted with caution due to the limited number of patients (Figure 2A).

Among genes related to EC pathogenesis, we found statistical increased expression in the group of Stage III-IV carcinomas and recurrences compared with healthy controls for *BRAF*, for the Wnt pathway component *CTNNB1*, for the TGF-beta superfamily cytokine *GDF15*, for *PIK3CA*, for NF-κB family member *RELA*, and for the RUNX transcription factor family member *RUNX1*, (Figure 2A). Regarding genes from hormone pathways, we found a significant increased expression of *STS* in advanced stage cases compared with controls (Figure 2B). Increased expression in both *ALDH* and *CD44* as genes related to stem cell features was observed in CTC immunoisolated from Stages III and IV high-risk EC patients and recurrences compared with controls (Figure 2C). The correlation between the levels of expression of these candidate biomarkers and the clinicopathological features of patients included in the study, indicated that the expression of *BRAF, PIK3CA, RELA, RUNX1* (all involved in endometrial carcinogenesis) and *CD44* (stem cell marker) was increased in CTC isolated from patients with the tumor infiltrating more than 50% of the myometrium (n = 18) compared with those isolated from patients having myometrial invasion below 50% of the myometrium (n = 13) (Additional file 3).

**Figure 3:**
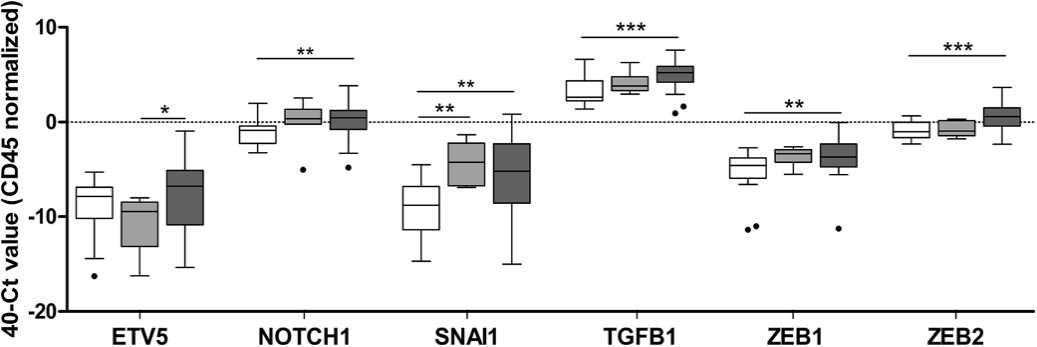
**Plasticity phenotype characterizes CTC in EC.** Almost all genes related to EMT assessed in CTC from high-risk EC patients presented significant expression compared to the background of unspecific immunoisolation. White boxes represent the gene expression levels in the group of healthy controls, light grey boxes those corresponding to FIGO Stages I and II EC patients while dark grey boxes those corresponding to FIGO Stages III-IV EC patients and recurrences. (Kruskal-Wallis test, *p < 0.05; **p < 0.01; ***p < 0.001).

More interestingly, almost the complete set of genes associated with EMT (*ETV5, NOTCH1, SNAI1, TGFB1, ZEB1* and *ZEB2*) were found to be specifically expressed in CTC from EC patients when compared to unspecific background from controls (Figure 3), with *LOXL2* achieving a value close to significance (p = 0.08; data not shown). We also found statistical increased expression levels in the EMT marker *ZEB2*, together with *RUNX1*, when we compared high-risk EC patients presenting recurrences (n = 15) or not (n = 18) (Additional file 3). Likewise, when we assessed the panel of CTC significantly expressed genes in a small series of matched primary endometrial carcinomas and affected lymph nodes (n = 6), the expression of the EMT related gene *ZEB2* demonstrated the best performance among a global tendency for an increased expression in lymph node metastasis compared to primary lesions, (p = 0.09; Additional file 4). No significant differences were found for any specific histology subtype regarding EMT markers, but we cannot exclude whether this could be due to the limited number of samples in the study or to the absence of differences in disseminated disease. We are currently increasing the number of paired matched samples in order to confirm these data. Overall, gene profiling on CTC isolated from high-risk EC patients suggested a plasticity phenotype characterized by genes of stem cell features and EMT, with potential in the clinical management of high-risk EC patients.

### ETV5 recapitulates the EMT phenotype characterizing CTC and impacts metastasis in an EC mouse model

**Figure 4:**
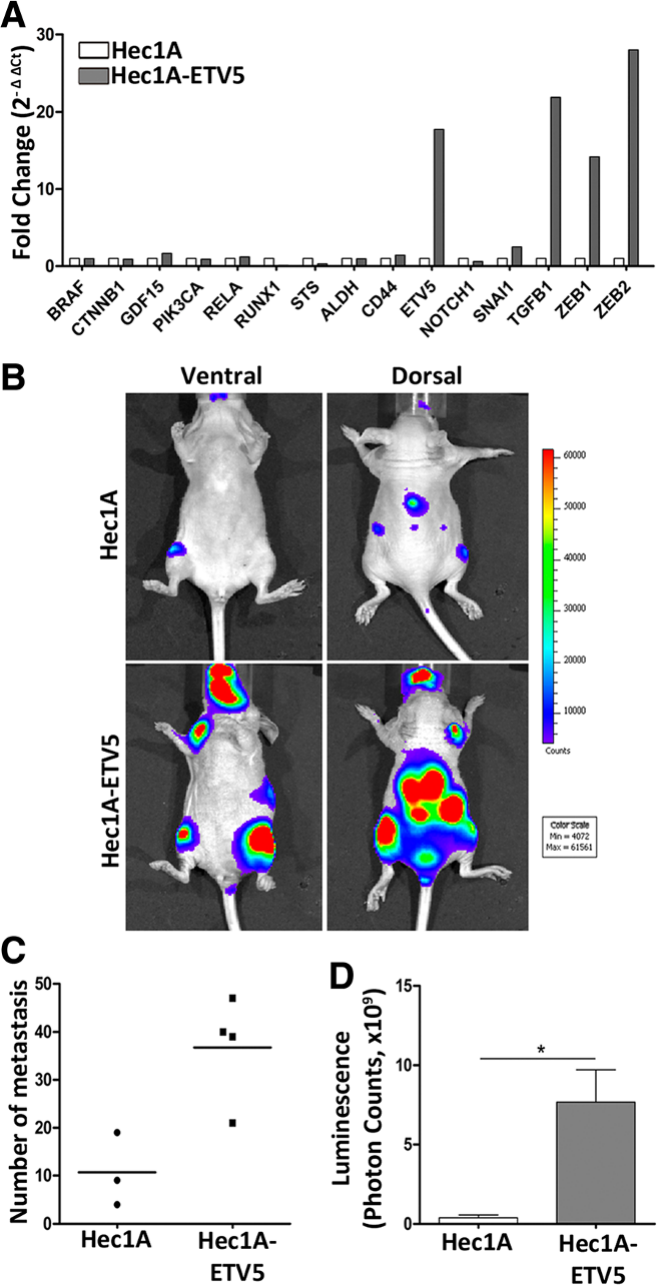
**ETV5 recapitulates the EMT phenotype found in CTC and the metastasis potential in an EC mouse model. (A)** CTC-gene expression profiling in Hec1A and Hec1A-ETV5 cell lines by RT-qPCR. The results were represented as the fold change in gene expression relative to *GAPDH* gene expression (2^-ΔΔCt^). **(B)** Representative luminiscence examples of athymic nude mice inoculated with Hec1A (upper panels) or Hec1A-ETV5 (lower panels) cells by intracardiac injection. Luminescence images were acquired for 1 min at ventral (left image) and dorsal (right image) positions. **(C)** Extent of dissemination evaluated as number of metastasis and **(D)** as luminescence quantification of metastasis.

ETV5 transcription factor, one of the candidate genes characterizing the CTC-plasticity phenotype, plays a major role in EC as a regulator of EMT [11, 12]. This led us to further asses the role of ETV5 in endometrial CTC and evaluate whether the invasive phenotype promoted by ETV5 during myometrial infiltration recapitulated the CTC phenotype observed in patients. To this end, the naïve EC cell line Hec1A and the cell line Hec1A over-expressing *ETV5* were used. As can be observed in Figure 4A, an increase in ETV5 expression resulted in the concomitant up-regulation of genes related to EMT, as observed in CTC. These results suggested that at least part of the plasticity phenotype observed in CTC in EC patients might be associated with the up-regulation of *ETV5* during myometrial invasion and confirmed the role of ETV5 as a master regulator of EMT.

We further investigated the impact of *ETV5* up-regulation and the consequent EMT phenotype in a mice model that reproduced the systemic dissemination, homing and generation of micrometastasis associated with CTC. For this, we compared the extent of metastasis resulting from the intracardiac injection of both naïve Hec1A cells (Figure 4B, upper panels), and *ETV5* over-expressing Hec1A cells (Figure 4B, lower panels), further modified to constitutively express the luciferase reporter gene and allow vital bioluminescent imaging of disseminating tumor cells. As shown in Figure 4B, the up-regulation of *ETV5* and the consequent recapitulation of the CTC-plasticity phenotype resulted in a more dramatic cell dissemination and metastatic spread, both in terms of pattern of metastasis localization as well as the number of metastasis foci (Figure 4C, D). In addition to reinforce the role of ETV5 during the initial events of metastasis in endometrial cancer, these results link the promotion of a plasticity phenotype in CTC with their capacity to metastasize.

## Discussion

We present in this study evidences for the presence of CTC in high-risk EC patients, and further characterized a molecular CTC-phenotype associated with plasticity and stemness features. The major clinical relevance of CTC is that the early detection in patients could be of use for the identification of candidate subjects needing additional systemic therapies after the resection of the primary tumor. Although the aim of these therapies is the prevention of metastasis, the selection of patients is nowadays based on the statistical risk of recurrences, which is not accurate in terms of over-treatment of patients with toxic agents or therapeutic procedures causing serious side effects, in addition to economic costs. An improved ability to target surgical and systemic therapies to well selected high-risk patient populations will increase the likelihood of benefits and decrease the side effects associated with un-necessary treatments [13]. In addition, the sequential assessment of CTC levels during treatment could provide information at early stages about the therapeutic efficacy of drugs. Likewise, the elimination of these CTC could represent an intermediate endpoint in clinical trials with antitumor drugs. Furthermore, the molecular and functional characterization of CTC will be determinant in the discovery of new molecular tumor markers and in the development of therapies specific for the process of tumor dissemination and metastasis. Overall, CTC represent a potent and promising tool in oncology and, although the clinical role of CTC as a prognosis factor is being recognized, there is still a need for more advanced and precise techniques of detection and robust clinic-pathologic correlations [14].

From our results, we can conclude that EpCAM-based immune-enrichment followed by RT-qPCR analysis is a reliable method to effectively isolate CTC from high-risk EC patients and to potentially distinguish high-risk from low-risk patients. Previous efforts to analyze and evaluate CTC in EC included the assessment of a six gene panel in blood samples [15]. Although this methodology represents a semi-quantitative evaluation of CTC, it demonstrated an added value compared to the gold standard CellSearch technology for the positive detection of CTC (see Additional file 1). In addition to the clinical utility of CTC as a surrogate marker in the management of high-risk and metastatic cancer patients, the challenge stands on the possibility of a therapeutic approach targeting these metastatic CTC with the aim of controlling and/or eradicating the source of recurrences. The advantage of combining CTC immunoisolation and RT-qPCR analysis consists in the possibility to identify and characterize biomarkers specific of this subpopulation of metastatic cells. This approach has the limitation to determine whether the increased levels in biomarker expression upon CTC immunoisolation is related to an enhanced expression or to an augmentation in the number of CTC. Nevertheless, in addition to their potential in terms of diagnosis/prognosis and follow-up of patients, these biomarkers provide with phenotypic clues on the biology of CTC that may be determinant in the identification of new therapeutic strategies aiming to specifically control and/or eradicate the metastatic dissemination in EC [16]. From a clinical perspective, the management of metastatic EC has been recently considered as a poly-chemotherapy adjuvant regimen for patients with high risk of relapse, and as a palliative regimen for patients with disseminated disease or with extrapelvic recurrence not responding to hormone treatment. In addition, endometrial carcinomas are considered as chemoresistant and the most active drugs (platinum salts, doxorubicin, anthracyclines and paclitaxel) present relative rate responses ranging from 25% in monotherapy to 57% in poly-chemotherapy, with median survival of 12–15 months. In this scenario, chemotherapy has shown limited utility and there is a clear need for the development of new rationale therapies focused on metastasis [17]. To this regard, our profiling of endometrial CTC has pointed out at a number of pathways relevant to the metastatic process and that could be targeted. For instance, the expression of *STS*, which is associated with higher availability of estrogens in tumor cells [18], can provide a growth advantage to CTC and its inhibition can be used to block such event and decrease the risk of recurrences. A second important marker highly expressed in CTC was *PIK3CA*, which confirms the role of this kinase pathway as a potential target in high-risk and metastatic disease [19–21].

A main feature observed in the molecular profiling of CTC in EC corresponded to the EMT phenotype, with almost all analyzed genes related to plasticity being significantly expressed in CTC (*ETV5, NOTCH1, SNAI1, TGFB1, ZEB1, ZEB2*). EMT is a dynamic process whereby epithelial cells lose polarity and cell-cell contacts, undergo dramatic remodeling of the cytoskeleton, acquire a migratory phenotype and a mesenchymal-like gene expression program. Both invasion and metastasis may be critically dependent on the acquisition by the incipient cancer cell of EMT features [22, 23]. Interestingly, both EMT and the PIK3CA pathway have been closely linked in the promotion of metastasis, particularly in EC [24, 25]. Our results also reinforce a role for ETV5 in the process of EMT and EC dissemination [11]. ETV5 up-regulation in Hec1A cells recapitulated in vitro the plasticity phenotype found in high-risk patients, and demonstrated an advantage in the promotion of metastasis in an in vivo mouse model that mimic CTC dissemination and homing.

Likewise, and concerning *ALDH* and *CD44* as stem-cell genes identified in this profiling, we observed a concordance between the presence of endometrial CTC and recurrent disease. Whether these CTC include a subpopulation of tumor cells with a stem cell-like phenotype (Cancer Initiating Cells [26]) or whole CTC population must be considered responsible for recurrences with more or less efficiency, has yet to be addressed. In addition to its plasticity phenotype, CTC triggering micrometastasis in the target organs must own the capacity to survive in the blood flow, to home and to regenerate a tumor mass with similar characteristics as the primary lesion in the tissue recipient of metastasis. Recent studies on CTC in breast cancer have demonstrated that a subset of isolated CTC express stem cell markers such as those analysed in our study [27, 28]. The evidence that tumor dissemination to the blood circulation is an early event and that the process of metastasis is an ineffective process with only a small number of CTC ending up in micrometastasis, support the hypothesis of a CTC phenotype with plasticity and stemness features with the capacity to develop metastasis [29]. This concept linking EMT and stem cell features in the process of tumor dissemination has also been addressed in EC [30], in association with a micro-RNA signature of EMT in endometrial carcinosarcoma mainly represented by the down-regulation of members of the miR-200 family [31]. Remarkably, the balanced expression of ZEB factors and miR-200 is considered as a molecular motor of cellular plasticity, in particular is a driving force for cancer progression towards metastasis by controlling the state of cancer stem cells [32, 33]. The results we obtained both in CTC and in paired carcinoma and lymph-node tissue samples demonstrating the potency of *ZEB2* within the CTC-phenotype reinforce the need of future investigations examining stem-like features in CTC to obtain relevant information about this cancer subpopulation responsible of metastases.

Finally, it should be note that although EpCAM expression was found consistent in primary endometrial carcinomas, its proposed modulation and eventual loss during EMT adds controversy to the efficiency of enrichment of CTC owning a plasticity phenotype [34]. To this regard, we analyzed the expression of EpCAM both in the epithelial endometrial cancer cell line HEC1A and its mesenchymal counterpart Hec1A-ETV5, and found similar levels of EpCAM expression irrespective of their EMT phenotype (Additional file 5). Moreover, EpCAM-based immunoisolation of these HEC1A and HEC1A-ETV5 cells lines rendered similar efficiencies (75% versus 64%, respectively). From these results, it seems reasonable to speculate that cells detaching from the primary lesion and incorporating into the blood stream recapitulate a metastable epithelial–mesenchymal phenotype that may be maintained during their way to those distant sites where this CTC will home and end up in the generation of micrometastasis. The dissociation of tumor cells from the epithelial layer and the penetration through the basement membrane into the adjacent connective tissue, are the initial events in the multistep process that characterizes metastasis [35]. We are additionally conducting further studies with other immunoisolating antigens.

## Conclusions

We present evidences for the presence of CTC in high-risk EC patients, and we propose a CTC-phenotype in EC associated with plasticity and stem cell features. Although this multicentre study conducted within the framework of ENITEC has the limitation of the number of samples, these promising data offer the opportunity to design new therapeutic strategies targeting metastatic disease, and a larger prospective study is aimed for the validation of the CTC-phenotype in high-risk EC.

## Methods

### Patient samples

Peripheral blood samples collected just before initiation of treatment from 34 EC patients and 27 controls were processed for CTC immunoisolation and accurate RNA extraction as described [7]. Patients participating in the study were surgically staged according to FIGO and recruited between March 2012 - October 2013 in Vall d’Hebron University Hospital (Barcelona, Spain), University Hospital of Santiago de Compostela (Santiago de Compostela, Spain), Arnau de Vilanova Hospital (Lleida, Spain), MD-Anderson Cancer Center Madrid (Madrid, Spain), Fundacion Dexeus (Barcelona, Spain) and Haukeland University Hospital (Bergen, Norway), and included high-risk endometrial carcinomas ranging from Grade 3 Stage IB carcinomas to metastatic Stage IV carcinomas and recurrences (Table 1). Control group included a set of 27 healthy women with absence of a previous cancer episode and with an age range similar to patients. Informed consent approved by the relevant ethical committee was signed by all patients. In addition, fresh-frozen tissue from primary tumor and paired affected lymphatic nodes from 6 EC patients were provided by Tumor Bank of the Vall d’Hebron University Hospital Biobank (Barcelona, Spain) with appropriate ethics approval.

### EpCAM immunohistochemistry

EpCAM expression was checked in whole paraffin-embedded sections of primary endometrial carcinomas from patients prospectively subjected to evaluation of CTC. Sections were dried for 1 h at 65°C before pre-treatment procedure of deparaffinization, rehydration and epitope retrieval in the Pre-Treatment Module, PT-LINK (DAKO) at 95°C for 20 min in Citrate buffer (10 mM), Low pH, endogenous peroxidase was blocked before staining with antibodies against Epithelial Related Antigen (clone MOC-31, dil. 1:50; DAKO, Denmark). After incubation, the reaction was visualized with the EnVision FLEX Detection Kit (DAKO) using diaminobenzidine chromogen as a substrate.

Positivity ranged from 75% to 100% with a mean of 93%.

### CTC immunoisolation and quantitative real-time polymerase chain reaction (RT-qPCR)

CTC immunoisolation with the CELLection™ Epithelial Enrich kit (Invitrogen, Dynal, Oslo, Norway), RNA extraction and RT-qPCR were carried out as previously described [7]. After EpCAM-based immune-enrichment of CTC following manufacturer’s protocol, RNA purification was performed with QiampViral kit (Qiagen, Valencia, CA, USA), optimized for very low cellularity samples. cDNA was synthesized using SuperScriptIII chemistry (Invitrogen, Carlsbad, CA, USA) according to the user’s guide and subjected to pre-amplification with TaqMan®PreAmp Master Mix kit (Applied Biosystems, Foster City, CA, USA) for 14 reaction cycles before proceeding to RTqPCR, to provide with optimal detection rates. TaqMan Gene Expression Assays (Applied Biosystems, Foster City, CA, USA) for 35 selected genes (Additional file 2; plus *GAPDH* as housekeeping gene and *CD45* as a marker of non-specific isolation) were used to measure the gene expression levels in CTC isolated from patients in comparison to the background of hematogenous cells unspecifically immunoisolated from the group of healthy controls. Values were analyzed using StepOne Software v.2.1 (Applied Biosystems, Foster City, CA, USA), normalized to *CD45* and represented as (40–ΔCt), whereby ΔCt = duplicate mean (CtTARGET – Ct*CD45*).

### Cell lines and cell culture

The human endometrial carcinoma cell lines Hec1A and Hec1A stably expressing the ETV5 transcription factor (Hec1A-ETV5) were maintained in McCoy’s 5A Medium (Gibco, Grand Island, NY, USA) supplemented with 10% FBS and 1% penicillin-streptomycin at 37°C in 5% CO_2_, Hec1A-ETV5 cells further selected with Geneticin (500 μg/ml; Gibco, Grand Island, NY, USA). These cells were previously generated and thoroughly characterized [11, 12].

To monitor non-invasively tumor grafts of Hec1A and Hec1A-ETV5 cells, these cells were infected with lentiviruses bearing pLenti CMV V5-LUC Blast (w567-1) (Addgene, Cambridge, MA, USA) to constitutively express the luciferase reporter gene, as previously described [36]. Stable infected cells expressing luciferase were selected with Blasticidine S HCl (3 μg/ml; Invitrogen, CA, USA).

### RNA extraction and real-time PCR

Paired frozen tumors and metastatic lymph node samples were disrupted and homogenized using a TissueLyser II (Qiagen, Valencia, CA, USA) and total RNA was extracted using the AllPrep DNA/RNA/Protein Mini Kit (Qiagen, Valencia, CA, USA) according to the manufacturer’s protocol. Alternatively, total RNA was isolated from Hec1A and Hec1A-ETV5 cells using High Pure RNA Isolation Kit (Roche Applied Science, Indianapolis, IN, USA) according to the manufacturer.

cDNA synthesis was carried out using MuLV reverse transcriptase Kit (Applied Biosystems, Foster City, CA, USA) following the instructions provided by the manufacturer. Real-time PCR was performed using Applied Biosystem 7500 Real-Time PCR Machine and data were analyzed with StepOne Software v.2.1 (Applied Biosystems, Foster City, CA, USA). *GAPDH* was used as an internal normalization control. The results were represented as fold change in gene expression relative to *GAPDH* gene expression (2^-ΔΔCt^).

### In vivo assay and bioluminescent imaging

Six Five-week-old female athymic Nude-Foxn1^nu^ mice were purchased from Harlan Laboratories (Indianapolis, IN). Mice were divided in 2 groups and either Hec1A or Hec1A-ETV5 stably expressing luciferase cells (5×10^5^ cells in 100 μl of sterile PBS) were inoculated into animals by intracardiac injection under 2% isoflurane/air anesthesia. A successful intracardiac injection was indicated on day 0 by images showing systemic bioluminescence distributed throughout the animal. Only mice with evidence of a satisfactory injection continued in the experiment. Three weeks after cells injection and before sacrifice, IVIS system (Xenogen Corporation) coupled to Living Imaging software 4.2 (Xenogen Corporation) were used to detect tumor metastases by bioluminescent imaging. For non-invasive bioluminescence tumour imaging, luciferin (Firefly Luciferin, Caliper Lifescience Corp, Hopkinton, MA, USA) was used as the substrate for the luciferase expressing tumor cells and injected intraperitoneally at a concentration of 150 mg/kg in PBS. Mice were housed and maintained under specific pathogen-free conditions and used in accordance with institutional guidelines approved by the Use Committee for Animal Care.

### Statistical analysis

Statistical analyses were conducted using SPSS (Chicago, version 15.00 for Windows) and GraphPad Prism 4.00 software (GraphPad Softwares Inc, San Diego, CA, USA). Mann–Whitney and Kruskal-Wallis non-parametric tests were used to determine the differences between conditions. For Kruskal-Wallis analysis we used Dunn’s post-test. Alternatively, Wilcoxon signed test was used to determine the differences in relative gene expression between paired frozen tumors and metastatic lymph node samples. Statistical significance was set at p < 0.05.

## Electronic supplementary material

Additional file 1: **CTC analysis using CellSearch technology.** (DOCX 897 KB)

Additional file 2: **Representative references and corresponding RTqPCR Taqman probes of genes covering the main biological functions assessed in CTC immunoisolated from high-risk EC patients.** (PDF 87 KB)

Additional file 3: **Satistical p-values corresponding to the correlation of CTC-gene expression with clinical and pathologic parameters of high-risk EC patients included in the study.** (DOCX 22 KB)

Additional file 4: **ZEB2 expression in paired samples of primary carcinoma (white box) and affected lymph nodes (grey box) from 6 EC patients.** ZEB2 increased expression in lymph node metastasis compared to primary lesions further reinforced the EMT phenotype in EC CTC. (PDF 90 KB)

Additional file 5: **EpCAM expression assessed in Hec1A and Hec1A-ETV5 cells using flow cytometry.** (PDF 244 KB)

## Abbreviations

EC: Endometrial cancer
CTC: Circulating tumor cells
ENITEC: European Network for Individualized Treatment in Endometrial Cancer
EMT: Epithelial-to-mesenchymal transition
FDA: Food and Drug Administration.
