# Supplementary material for: Molecular profiling of circulating tumor cells links plasticity to the metastatic process in endometrial cancer
Source: Mol Cancer. 2014 Sep 27;13:223. doi: 10.1186/1476-4598-13-223 (PMC4190574; doi:10.1186/1476-4598-13-223)
Supplement: Supplementary file 1 — Additional file 1: CTC analysis using CellSearch technology. (DOCX 897 KB) [file 12943_2014_1426_MOESM1_ESM.docx]

**CTC analysis using CellSearch technology**

In parallel to CTC immunoisolation and GAPDH/CD45 RT-qPCR quantification, a second independent set of samples from 32 high-risk EC patients was processed by CellSearch (Janssen Diagnostics, SouthRaritan, NJ, USA) technology which combines immunoenrichment and immunofluorescence for the detection of CTC, and received Food and Drug Administration (FDA) clearance for the use as an aid in the monitoring of metastatic breast, colorectal, and prostate cancer [8, 9, 10].

**Clinical and pathologic characteristics of 32 high-risk EC patients included in the study of CTC using CellSearch technology.**

| **n (%)** | **n (%)** |
| --- | --- |
| Age (years)  Mean 69,4 (49-88) | Lymphovascular invasion  Positive 9 (28,1)  Negative 17 (53,1)  Unknown 6 (18,8) |
| FIGO stage  I 11 (34,4)  II 2 (6,3)  III 11 (34,4)  IV 6 (18,6)  Unknown 2 (6,3) | Lymph node metastasis  Positive 16 (50)  Negative 12 (37,5)  Unknown 4 (12,5) |
| Histology  Endometrioid 20 (62,5)  Serous 10 (31,2)  Clear cell 2 (6,3) | Recurrence  No 20 (60)  Yes 12 (40) |
| Grade  Well differentiated (G1) 3 (9,4)  Moderately differentiated (G2) 7 (21,8)  Poorly differentiated (G3) 22 (68,8) | First treatment  Radiotherapy 4 (12,5)  Chemotherapy 4 (12,5)  None 24 (62,5)  Unknown 4 (12,5) |
| Myometrial invasion  <50% 9 (28,1)  >50% 21 (65,7)  Unknown 2 (6,2) |  |

Unknown values were considered as missing and therefore excluded from the statistical analysis.

Briefly, samples were drawn into 10ml evacuated blood draw tubes (CellSave Preservative tubes, Janssen Diagnostics), maintained at room temperature, and processed within 96 hours of collection. The semiautomated CellSearch technology performed an immunomagnetic enrichment of nucleated cells expressing EpCAM from 7.5ml of blood, and a fluorescent labeling with DAPI nuclear marker, CD45-APC for cells of hematogenous origin, and CK-PE cytokeratin staining for epithelial cells. Images of stained cells were acquired by a semiautomated fluorescence microscopy system (Celltracks Analyzer II), and two experimented reviewers selected the CTC, according to a round-oval morphology, nucleated (DAPI+ and ≥ 4µm), lacking CD45 and expressing CK-PE (CK8, 18 and 19) cells from the gallery of objects proposed by the system.

CTC counting indicated that 21.9% (n=7) of patients had positivity for CTC identification with a range of 1 to 10 CTC. Interestingly, patients with high grade tumors were those with more CTC compared to low grade tumors (31.5% in grade 3 tumors vs. 10% in grade 1 or 2 tumors, data not shown), and CTC levels were higher in patients with metastatic FIGO Stage IV compared to earlier stages.


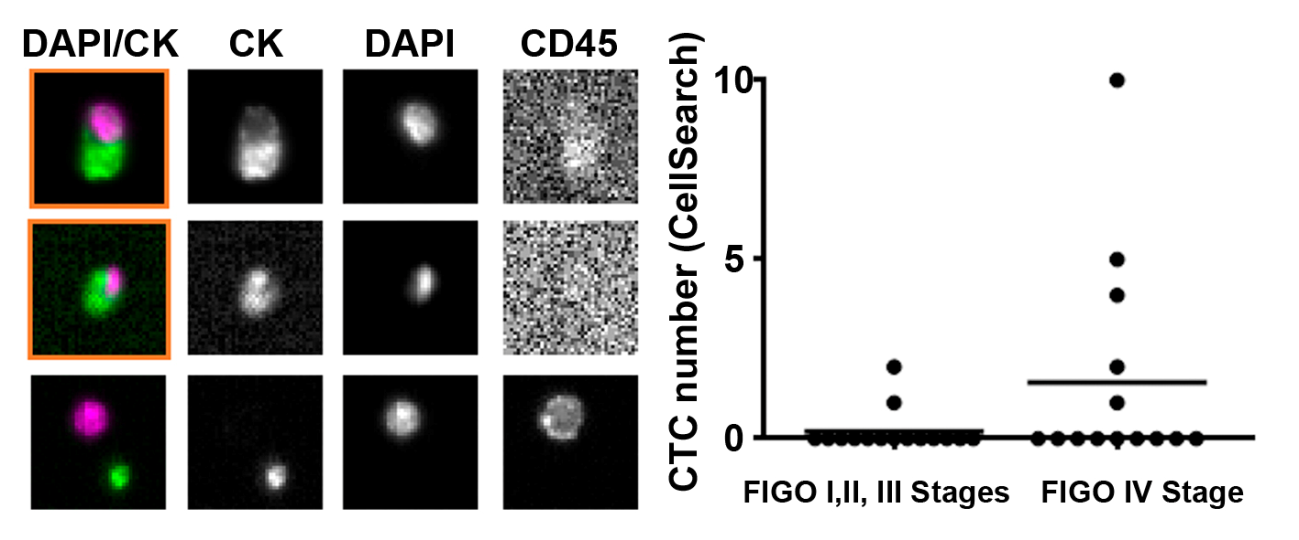


**Quantification of CTC based on CellSearch technology.** Representative examples of CTC are shown in upper and middle left panels while a non-CTC of hematopoietic origin is shown in the lower left panel; images are merge of cytokeratin (CK) staining indicative of epithelial origin, DAPI for nucleated cells and CD45 as marker of hematopoietic cells. The quantification of CTC with CellSearch technology in non-metastatic high-risk versus metastatic and recurrent EC patients is shown in the right panel, demonstrating the presence of CTC almost restricted to FIGO Stage IV patients.

We finally analyzed in a subset of patients included in the CellSearch study, the RT-qPCR expression levels of the panel of biomarkers (n=22). Clinical and pathologic data of this subset of patients is as follows:

|  | **n (%)** |  | **n (%)** |
| --- | --- | --- | --- |
|  |  |  |  |
| Age (years) |  | Lymphovascular invasion |  |
| Mean | 68.63 | Positive | 6 (27.3) |
|  |  | Negative | 15 (68.2) |
|  |  | Unknown | 1 (4.5) |
| FIGO Stage |  | Lymph node metastasis |  |
| I | 9 (40.9) | Positive  Negative  Unknown | 10 (45.5) |
| II | 1 (4.5) | Negative | 12 (54.5) |
| III | 8 (36.4) |  |  |
| IV | 4 (18.2) |  |  |
| Histology |  | Recurrence |  |
| Endometrioid | 15 (68.2) | Yes | 7 (31.8) |
| Serous | 7 (31.8) | No | 15 (68.2) |
| Grade |  | First treatment |  |
| G1 | 2 (9.1) | RT | 4 (18.2) |
| G2 | 4 (18.2) | CTX | 1 (4.5) |
| G3 | 16 (72.7) | None | 17 (77.3) |
|  |  | Unknown | 1 (4.5) |
| Myometrial invasión |  |  |  |
| <50% | 6 (27.3) |  |  |
| >50% | 15 (68.2) |  |  |
| Unknown | 1 (4.5) |  |  |

Unknown values were considered as missing and therefore excluded from the statistical analysis.

Among genes included in the panel, we found significant higher levels of *GDF15* (p=0,02) and the EMT related markers *ETV5* and *TGFB1* in patients with 1 or more CTC quantified by CellSearch, further indicating the relevance of the plasticity phenotype in the process of endometrial cancer dissemination and metastasis.

**
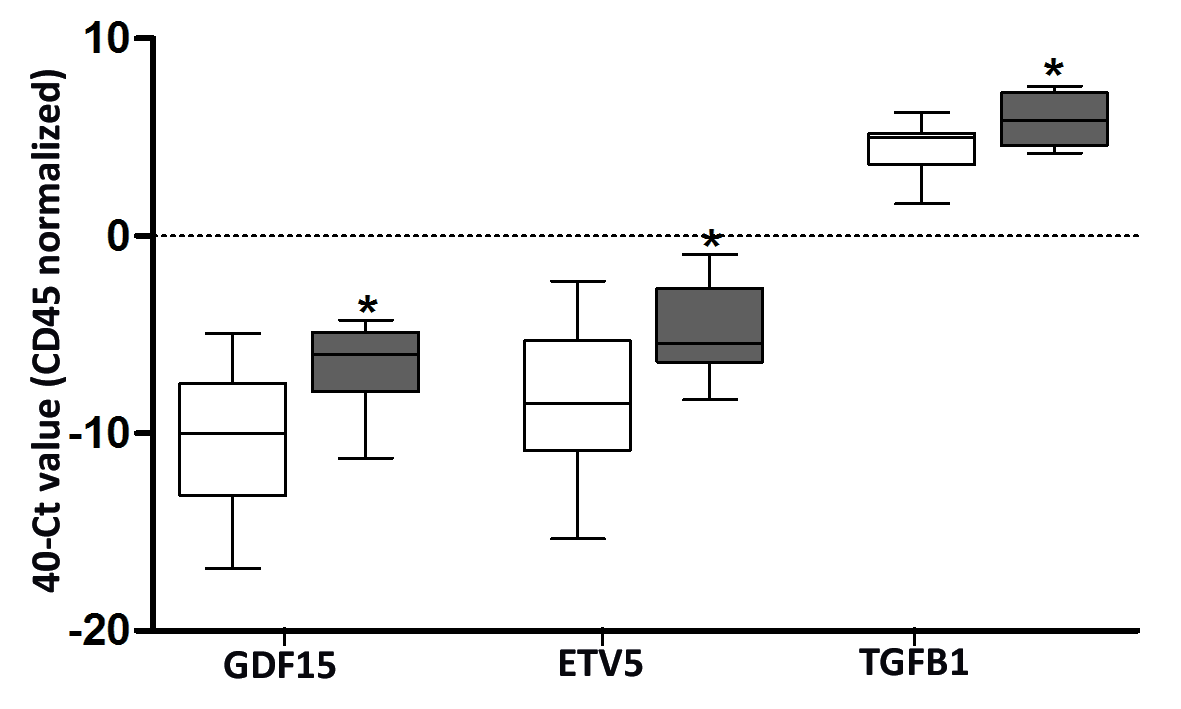
**

High GDF15, ETV5 and TGFB1 expression correlated with positive detection of CTC by CellSearch technology in high-risk EC patients. White and grey boxes represent the levels of expression in the group of patients negative and positive for CTC detection by CellSearch technology, respectively. (Mann-Whitney test, *p<0.05).

Globally, these results confirmed the presence of CTC in high-risk endometrial cancer patients. The relatively low percentage of positive CTC patients, mainly concentrated in Stage IV disseminated disease, could be due to the stringent criteria used by CellSearch. CTC identifying with CellSearch technology is mainly based on the size of the cytokeratin (CK8, 18+, and/or 19+) signal that should be ≥ 4µm and on the location of the DAPI signal, which should be at least 50% inside the cytokeratin signal. According to these parameters, we hypothesize that CellSearch technology might be discarding tumor cell fragments and multivesicular bodies carrying RNA as a cargo. Furthermore, significant proportion of CTC that express other cytokeratins different than CK8, 18 and/or 19 are also rejected but, on the contrary, may be isolated with EpCAM magnetic-beads and contribute to the RT-qPCR gene expression levels of the candidate genes. We are currently evaluating the possible contribution of multivesicular bodies purified from blood samples of high-risk EC patients to the CTC-phenotype.
