# Supplementary material for: Molecular profiling of circulating tumor cells links plasticity to the metastatic process in endometrial cancer
Source: Mol Cancer. 2014 Sep 27;13:223. doi: 10.1186/1476-4598-13-223 (PMC4190574; doi:10.1186/1476-4598-13-223)
Supplement: Supplementary file 2 — Additional file 2: Representative references and corresponding RTqPCR Taqman probes of genes covering the main biological functions assessed in CTC immunoisolated from high-risk EC patients. (PDF 87 KB) [file 12943_2014_1426_MOESM2_ESM.pdf]

Additional File 2. Representative references and corresponding RTqPCR Taqman Probes of genes covering the main biological functions assessed in CTC immunoisolated from high-risk EC patients.

	GENE	REFERENCES	TAQMAN PROBE
SIGNALLING PATHWAYS RELATED TO ENDOMETRIAL CANCER	BRAF	- Moreno-Bueno G. et al., Clin Cancer Res. 2006; 12(12):3865	Hs00269944_m1
	CTNNB1	- Nout RA. et al. Gynecol Oncol 2012;126(3):466-73.	Hs00355049_m1
	ERBB2	- Yeremian A. et al., Oncogene. 2013; 32(4):403-13.	Hs01001580_m1
	FGFR2	- Dutt A. et al. Proc Natl Acad Sci USA 2008; 105(25):8713-7.	Hs01552926_m1
	GDF15	- Staff AC. et al. Clin Cancer Res 2011;17(14):4825-33.	Hs00171132_m1
	IDO	- de Jong RA. et al. Gynecol Oncol 2012;126(3):474-80.	Hs00984148_m1
	MTOR	- Slomovitz BM & Coleman RL. Clin Cancer Res. 2012; 18(21):5856-64	Hs00234508_m1
	p53	- Nout RA. et al. Gynecol Oncol 2012;126(3):466-73.	Hs01034249_m1
	PIK3CA	- Salvesen HB et al. Lancet Oncol. 2012; 13(8):e353-61.	Hs00907957_m1
	PTEN	- Matias-Guiu X & Prat J. Histopathology. 2013; 62(1):111-23.	Hs02621230_s1
	PTGS2	- Jongen VH. et al. Int J Gynecol Cancer 2009; 19(4):670-6.	Hs01573471_m1
	RELA	- Pallares J. et al. J Pathol 2004;204(5):569-77.	Hs00153294_m1
	RUNX1	- Doll A. et al. Int J Cancer 2009;125(2):257-63.	Hs00231079_m1
	STMN1	- Salvesen HB et al. Proc Natl Acad Sci USA. 2009;106(12):4834-9.	Hs00606370_m1
	TERT	- Prescott et al., Cancer. 2010; 116(18):4275-82	Hs00972656_m1
	VIL1	- Nakamura E. et al. Cancer Biol Ther 2011;12(3):181-90.	Hs00200229_m1
	ZWINT	- Kasuboski JM et al., Mol Biol Cell. 2011 Sep;22(18):3318-30.	Hs00199952_m1
HORMONE PATHWAYS	CYP19	- Jongen VH. et al. Int J Gynecol Cancer 2009; 19(4):670-6.	Hs00903413_m1
	ESR1	- Vandenput I. et al.Int J Gynecol Cancer 2011; 21(2):316-22.	Hs00174860_m1
	ESR2	- Vandenput I. et al.Int J Gynecol Cancer 2011; 21(2):316-22.	Hs01100353_m1
	GPER	- Krakstad C. et al. Br J Cancer 2012;106(10):1682-8.	Hs00173506_m1
	HSD17B1	- Cornel KM. et al. J Clin Endocrinol Metab 2012; 97(4):E591-601.	Hs00166219_g1
	PGR	- Vandenput I. et al.Int J Gynecol Cancer 2011; 21(2):316-22.	Hs01556702_m1
	STS	- Purohit A & Foster PA. J Endocrinol. 2012 Feb;212(2):99-110.	Hs00996676_m1
	TFF1	- Dassen H. et al., Am J Pathol. 2010 Nov;177(5):2495-508.	Hs00907239_m1
STEM CELL	ALDH	- Rutella S. et al., Clin Cancer Res. 2009 Jul 1;15(13):4299-311.	Hs01007998_m1
	CD133	- Friel AM. Et al., Reprod Biol Endocrinol. 2010; 8:147.	Hs01009250_m1
	CD44	- Cervello I et al., Int J Gynecol Pathol. 2011 Jul;30(4):317-27.	Hs01075861_m1
EMT MARKERS	ETV5	- Colas E. et al. Oncogene 2012;31(45):4778-88.	Hs00231790_m1
	LOXL2	- Cano A. et al. Future Oncol 2012;8(9):1095-108.	Hs00158757_m1
	NOTCH1	- Mitsuhashi Y. et al. Histopathology 2012;60(5):826-37.	Hs01062014_m1
	SNAI1	- Montserrat N. et al. Hum Pathol. 2012 May;43(5):632-43.	Hs00195591_m1
	TGFB1	- Muinelo-Romay L. et al. Mol Cancer Ther 2011;10(8):1357-66.	Hs00998133_m1
	ZEB1	- Colas E. et al., Clin Transl Oncol. 2012 Oct;14(10):715-20.	Hs01566407_m1
	ZEB2	- Castilla MA. et al. J Pathol 2011; 223(1):72-80.	Hs00207691_m1
