# Supplementary material for: Molecular profiling of circulating tumor cells links plasticity to the metastatic process in endometrial cancer
Source: Mol Cancer. 2014 Sep 27;13:223. doi: 10.1186/1476-4598-13-223 (PMC4190574; doi:10.1186/1476-4598-13-223)
Supplement: Supplementary file 3 — Additional file 3: Satistical p-values corresponding to the correlation of CTC-gene expression with clinical and pathologic parameters of high-risk EC patients included in the study. (DOCX 22 KB) [file 12943_2014_1426_MOESM3_ESM.docx]

**Additional File 3.** Satistical p-values corresponding to the correlation of CTC-gene expression with clinical and pathologic parameters of high-risk EC patients included in the study (see Table I).

| CLINICAL-PATHOLOGIC FEATURES | Type I (n=19) vs  Type II (n=15) | G1- G2 (n=10) vs G3 (n=21) | Myometrial invasion <50% (n=13) vs >50% (n=18) | Lymphovascular invasion positive (n=6) vs negative (n=15) | Lymph node affectation positive (n=12) vs negative (n=20) | FIGO Stages*  I-II-III (n=28) vs Stage IV (n=6) | Recurrence **  no (n=18)  vs yes  (n=15) |
| --- | --- | --- | --- | --- | --- | --- | --- |
| BRAF | 0, 567 | 0,205 | **0,002** | 0,533 | 0,139 | **0,008** | 0,202 |
| GDF15 | 0,140 | 0,118 | 0,631 | 0,533 | 0,938 | 0,145 | 0,901 |
| PIK3CA | 0,395 | 0,642 | **0,003** | 0,119 | 0,969 | **0,037** | 0,067 |
| RELA | 0,742 | 0,237 | **0,018** | 0,484 | 0,726 | **0,029** | 0,901 |
| RUNX1 | 0,795 | 0,447 | **0,007** | 0,350 | 0,613 | 0,074 | **0,040** |
| STS | 0,903 | 0,331 | 0,118 | 0,533 | 0,392 | 0,982 | 0,244 |
| ALDH | 0,768 | 0,899 | 0,093 | 0,186 | 0,613 | 0,612 | 0,682 |
| CD44 | 0,849 | 0,735 | **0,041** | 0,484 | 0,613 | 0,204 | 0,681 |
| CTNNB1 | 0,275 | 0,108 | 0,085 | 0,533 | 0,484 | 0,238 | 0,957 |
| ETV5 | 0,260 | 0,833 | 0,810 | 0,697 | 0,139 | 0,053 | 0,135 |
| NOTCH1 | 0,876 | 0,447 | 0,200 | 0,392 | 0,969 | 0,439 | 0,509 |
| SNAI1 | 0,822 | 0,398 | 0,230 | 0,876 | 0,755 | 0,522 | 0,682 |
| TGFB1 | 0,206 | 0,735 | 0,631 | 0,586 | 0,969 | 0,066 | 0,073 |
| ZEB1 | 0,477 | 0,310 | 0,795 | 0,312 | 0,572 | 0,836 | 0,325 |
| ZEB2 | 0,340 | 0,673 | 0,072 | 0,392 | 0,969 | 0,082 | **0,011** |

* FIGO stage at diagnosis, ** recurrence at the moment of CTC analysis
