# Supplementary material for: Molecular profiling of circulating tumor cells links plasticity to the metastatic process in endometrial cancer
Source: Mol Cancer. 2014 Sep 27;13:223. doi: 10.1186/1476-4598-13-223 (PMC4190574; doi:10.1186/1476-4598-13-223)
Supplement: Supplementary file 4 — Additional file 4: ZEB2 expression in paired samples of primary carcinoma (white box) and affected lymph nodes (grey box) from 6 EC patients. ZEB2 increased expression in lymph node metastasis compared to primary lesions further reinforced the EMT phenotype in EC CTC. (PDF 90 KB) [file 12943_2014_1426_MOESM4_ESM.pdf]

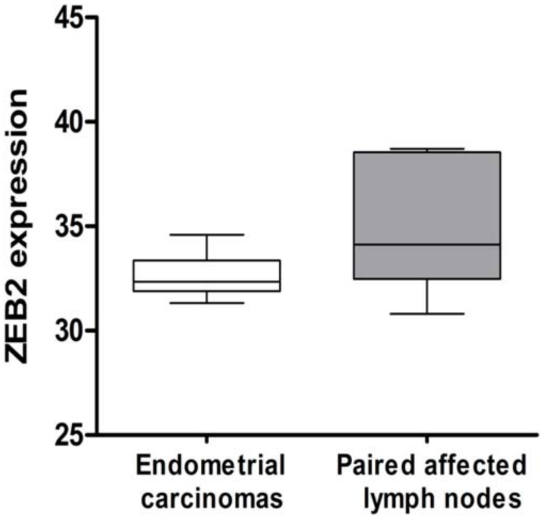

Additional File 4. ZEB2 expression in paired samples of primary carcinoma (white box) and affected lymph nodes (grey box) from 6 EC patients. ZEB2 increased expression in lymph node metastasis compared to primary lesions further reinforced the EMT phenotype in EC CTC.
