# Supplementary material for: Molecular profiling of circulating tumor cells links plasticity to the metastatic process in endometrial cancer
Source: Mol Cancer. 2014 Sep 27;13:223. doi: 10.1186/1476-4598-13-223 (PMC4190574; doi:10.1186/1476-4598-13-223)
Supplement: Supplementary file 5 — Additional file 5: EpCAM expression assessed in Hec1A and Hec1A-ETV5 cells using flow cytometry. (PDF 244 KB) [file 12943_2014_1426_MOESM5_ESM.pdf]

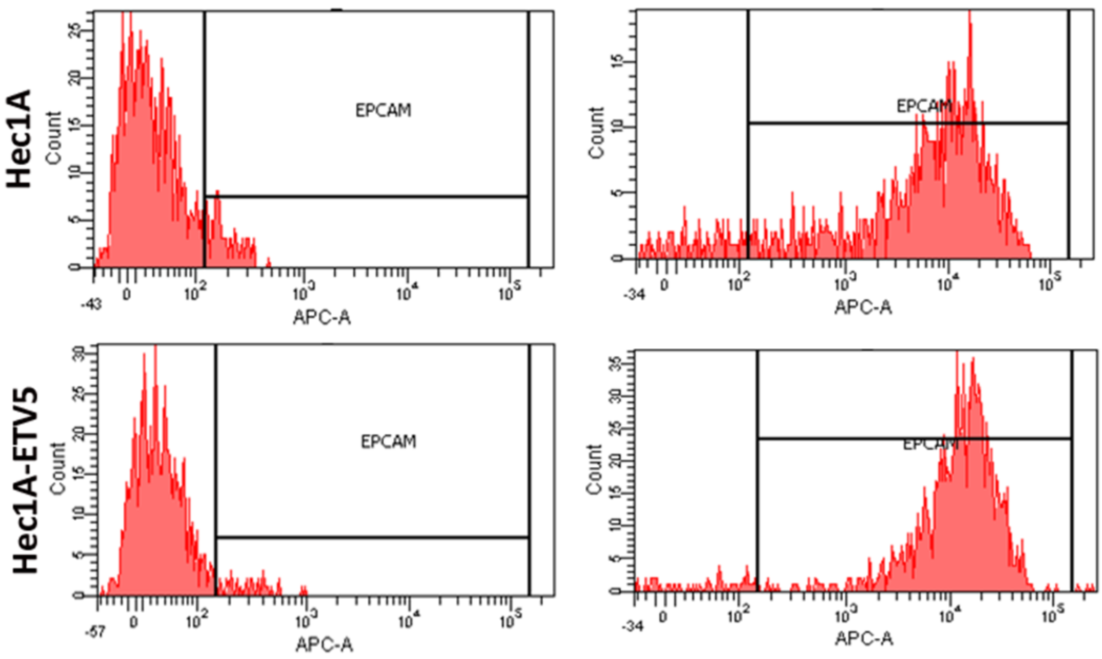

Additional File 5. EpCAM expression assessed in Hec1A and Hec1A-ETV5 cells using flow cytometry. $1x10^6$ Hec1A and Hec1A-ETV5 cells were fixed in 100µl of formaldehyde 3,7-4% buffer (Panreac) at room temperature for 10 min. After washing with PBS-Tween 0,1%-BSA 2%, the cells were incubated with EpCAM antibody in PBS-Tween 0,1%-BSA2% (2,5mg/ml; Biolegend) at 4°C for 1 h. Cells were washed in PBS and incubated with anti-mouse Alexa647 antibody (Abcam) at 4°C for 30 min. Cells incubated only with the secondary antibody served as negative control for non-specific antibody binding. The samples were analyzed by flow cytometry (FACSAria, BD Biosciences). Cells incubated only with Alexa647antibody (upper and lower left panels) or with anti-EpCAM antibody followed Alexa647antibody (upper and lower right panels).
